# Marine nutraceuticals from Mexican Pacific Sargassum targeting oxidative stress and inflammation in age-related macular degeneration

**DOI:** 10.3389/fnut.2026.1888072

**Published:** 2026-07-24

**Authors:** Lidianys Maria Lewis-Luján, Annette Pulcherie Iloki Lewis, Diego Emmanuel Guerrero Magaña, Clara Rosalia Alvarez Chavez, Mikhail A. Osadchuk, Maxim V. Trushin, Juan Carlos Galvez Ruiz, Judas Tadeo Vargas Durazo, Cinthia Jhovanna Perez Martinez, Sergio Trujillo López, Simon Bernard Iloki-Assanga

**Affiliations:** 1 Department of Biological Chemical Sciences, Sonora University, Hermosillo, Sonora, Mexico; 2 Department of Medicine and Health Sciences, University of Sonora, Hermosillo, Sonora, Mexico; 3 Federal State Autonomous Education Institution of Higher Training, First Sechenov Moscow State Medical University, Moscow, Russia; 4 Department of Biology, Institute of Fundamental Medicine and Biology, Kazan Federal University, Kazan, Russia

**Keywords:** age-related macular degeneration, fucoidans, fucoxanthin, oxidative stress, retinal pigment epithelium, Sargassum

## Abstract

Age-related macular degeneration (AMD) is a multifactorial retinal neurodegenerative disease characterized by oxidative stress, chronic inflammation, retinal pigment epithelium (RPE) dysfunction, and progressive central vision loss. Marine-derived bioactive compounds from Mexican Pacific Sargassum species have emerged as promising nutraceutical candidates due to their antioxidant, anti-inflammatory, and cytoprotective properties. This narrative review critically examines the nutritional composition and pharmacologically relevant bioactive constituents of Mexican Pacific Sargassum, with emphasis on fucoxanthin, fucoidans, phlorotannins, and polyunsaturated fatty acids. Particular attention is given to their molecular mechanisms of action in AMD-related pathways, including modulation of oxidative stress, Nrf2/HO-1 signaling, NF-κB-mediated inflammation, VEGF-associated angiogenesis, mitochondrial dysfunction, and apoptosis in retinal cells. Current evidence from preclinical retinal models suggests that these compounds may exert protective effects against AMD progression through multipronged regulation of redox and inflammatory pathways. Additionally, major translational challenges related to bioavailability, extraction standardization, safety, and the absence of AMD-specific clinical trials are critically discussed. Overall, Mexican Pacific Sargassum represents a promising yet underexplored source of marine bioactives with potential applications in the development of nutraceutical strategies targeting retinal degeneration and AMD.

## Graphical Abstract

**Figure:**
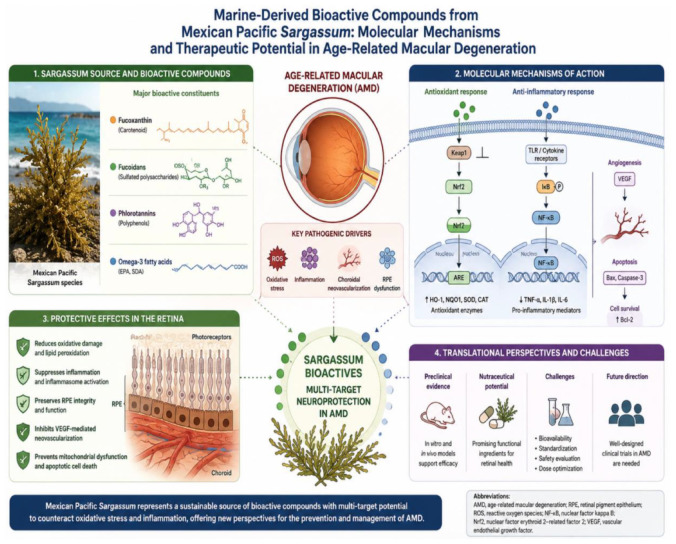
Overview of the principal pathogenic mechanisms involved in age-related macular degeneration (AMD), highlighting the multitarget protective actions of bioactive compounds from Mexican Pacific Sargassum. Fucoxanthin, fucoidans, phlorotannins, and omega-3 fatty acids modulate oxidative stress, inflammation, mitochondrial dysfunction, angiogenesis, and apoptosis, thereby contributing to retinal pigment epithelium protection and representing promising nutraceutical candidates for AMD prevention and management.

## Highlights

- Mexican Pacific Sargassum contains bioactive compounds with antioxidant, anti-inflammatory, and neuroprotective properties relevant to AMD.
- Fucoxanthin, fucoidans, and phlorotannins modulate oxidative stress, retinal inflammation, and RPE dysfunction.
- Sargassum-derived bioactives regulate Nrf2/HO-1, NF-κB, VEGF, and apoptosis-related signaling pathways.
- Preclinical evidence supports the therapeutic potential of marine bioactives in retinal degeneration and AMD progression.
- Bioavailability, extraction standardization, and lack of clinical trials remain major translational challenges.

## 1 Introduction

Age-related macular degeneration (AMD) is a progressive multifactorial retinal disorder and one of the leading causes of irreversible visual impairment and blindness among elderly populations worldwide, profoundly affecting quality of life, independence, and socioeconomic wellbeing (1, 2). The disease is characterized by degeneration of the retinal pigment epithelium (RPE), photoreceptor dysfunction, drusen accumulation, mitochondrial alterations, and, in advanced stages, geographic atrophy or choroidal neovascularization. Increasing evidence indicates that oxidative stress, chronic para-inflammation, dysregulated angiogenesis, mitochondrial dysfunction, and genetic susceptibility are central mechanisms driving AMD pathogenesis (3, 4). Due to these interconnected pathogenic processes, AMD has also been associated with systemic age-related disorders, including hypertension, obesity, diabetes mellitus, coronary heart disease, chronic kidney disease, and neurodegenerative conditions, highlighting its relevance as a component of broader multisystem aging pathology (Table 1).

**TABLE 1:** Major bioactive compounds from Mexican Pacific Sargassum and their proposed molecular mechanisms in age-related macular degeneration (AMD).

Bioactive compound	Major molecular targets/pathways	Proposed retinal effects in AMD	Experimental evidence	Translational relevance	References
Fucoxanthin	Nrf2/HO-1 activation; ROS suppression; inhibition of NF-κB signaling	Reduction of oxidative stress, preservation of RPE integrity, attenuation of apoptosis and mitochondrial dysfunction	ARPE-19 cells, retinal oxidative stress models	Potential nutraceutical candidate for oxidative retinal disorders	(10–12)
Fucoidans	VEGF inhibition; anti-inflammatory signaling; modulation of cytokine release	Anti-angiogenic effects, suppression of retinal inflammation, reduction of pathological neovascularization	*In vitro* RPE and angiogenesis models	Potential adjunctive strategy targeting neovascular AMD	(13–15)
Phlorotannins	ROS scavenging; mitochondrial protection; anti-inflammatory activity	Prevention of oxidative DNA damage and apoptosis in retinal cells	ARPE-19 oxidative stress models	Oxidative stress models potential neuroprotective and antioxidant retinal agent	(16–18)
Omega-3 fatty acids (EPA/DHA)	Modulation of inflammatory mediators and membrane stability	Retinal protection and modulation of inflammatory signaling	Clinical and nutritional studies	Limited benefit demonstrated in AREDS2 supplementation	(19, 20)
Polyphenolic compounds	Regulation of oxidative stress and inflammatory pathways	Reduction of lipid peroxidation and cellular oxidative injury	Preclinical antioxidant studies	Potential complementary nutraceutical approach in AMD	(7, 21)

Oxidative stress plays a pivotal role in retinal degeneration because the retina is characterized by exceptionally high oxygen consumption, continuous exposure to visible light, elevated concentrations of polyunsaturated fatty acids, and intense mitochondrial metabolic activity. Excessive generation of reactive oxygen species (ROS) promotes lipid peroxidation, mitochondrial dysfunction, inflammatory signaling, and apoptosis in retinal cells, particularly within the RPE. Concurrently, chronic low-grade inflammation involving NF-κB activation, complement dysregulation, inflammasome signaling, and pro-inflammatory cytokine release further contributes to retinal injury and AMD progression (3, 4). Moreover, the accumulation of β-amyloid deposits and cholesterol-rich drusen within the retina has led to the hypothesis that AMD may represent a manifestation of accelerated retinal and brain aging processes (5).

**FIGURE 1:**
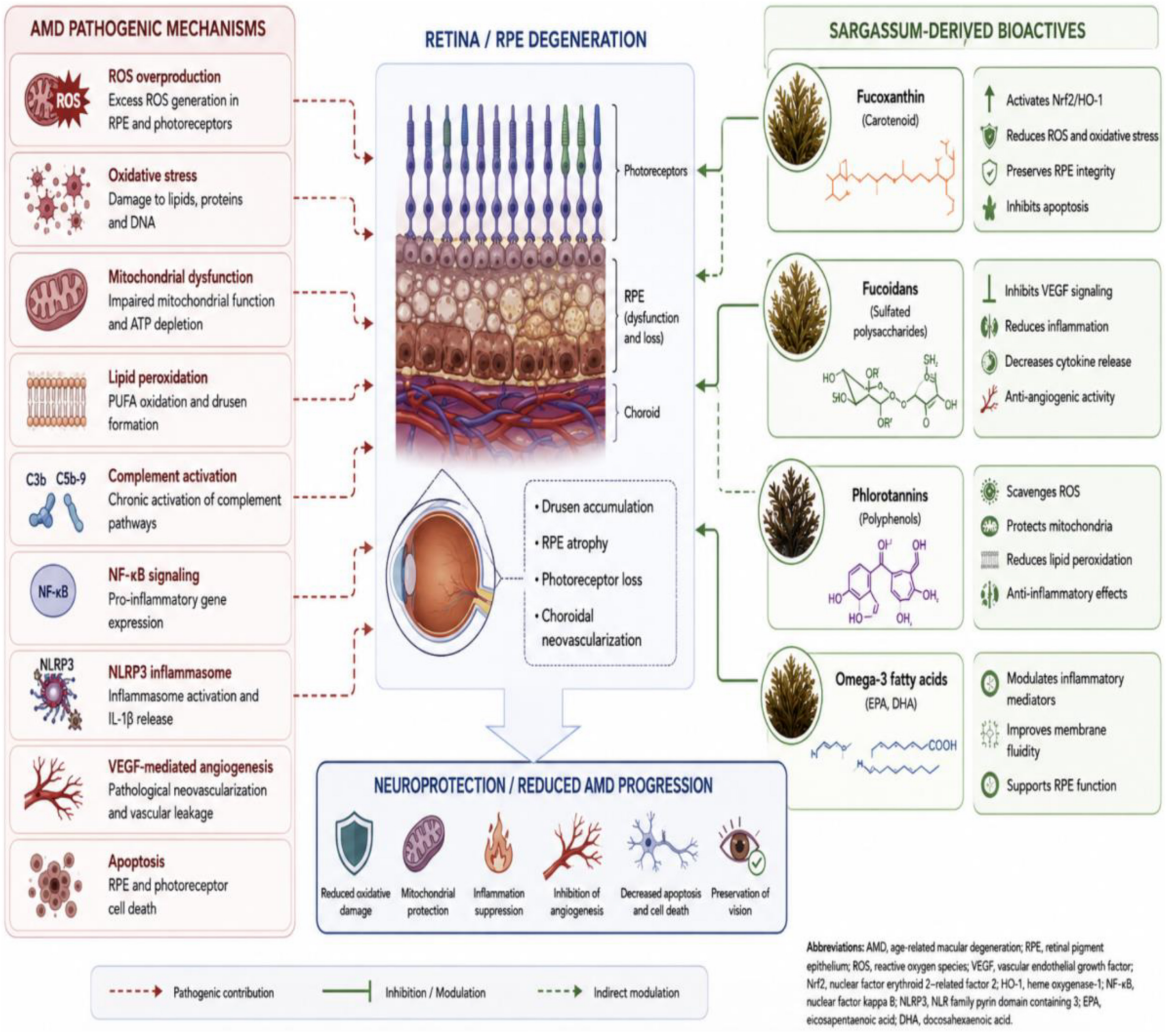
Integrated pathogenic mechanisms in AMD and potential molecular targets of Sargassum-derived bioactive compounds.

Figure 1 summarizes the major pathogenic mechanisms involved in AMD progression, including oxidative stress, chronic inflammation, mitochondrial dysfunction, angiogenesis, and retinal pigment epithelium degeneration, as well as the potential molecular targets modulated by Sargassum-derived bioactive compounds.

In recent years, increasing attention has been directed toward naturally derived bioactive compounds as potential adjunctive strategies for retinal protection and AMD prevention. Plant-derived polyphenols, carotenoids, and other nutraceutical compounds have demonstrated antioxidant, anti-inflammatory, mitochondrial-protective, and neuroprotective properties capable of modulating key molecular pathways implicated in AMD pathophysiology (6, 7). In particular, marine algae have emerged as highly promising sources of structurally diverse bioactive molecules with potent pharmacological activities, including antioxidative, anti-inflammatory, anti-apoptotic, and cytoprotective effects (8, 9). Brown seaweeds of the genus Sargassum contain several biologically relevant compounds, including fucoxanthin, fucoidans, phlorotannins, sulfated polysaccharides, and polyunsaturated fatty acids, many of which have demonstrated protective effects in experimental models of oxidative stress and inflammation.

The major bioactive compounds identified in Mexican Pacific Sargassum and their proposed molecular targets and retinal protective mechanisms relevant to AMD are summarized in Table 1.

Among marine resources, Mexican Pacific Sargassum species remain comparatively understudied despite their considerable biochemical diversity and potential nutraceutical value. Emerging evidence suggests that these marine-derived compounds may modulate critical pathogenic mechanisms associated with AMD, including oxidative stress, inflammatory signaling, angiogenesis, mitochondrial dysfunction, and apoptosis in retinal cells. However, important translational challenges remain, including limited clinical evidence, bioavailability constraints, extraction standardization, and safety evaluation.

Therefore, this narrative review aims to provide a comprehensive and updated overview of the nutritional composition, pharmacologically relevant bioactive compounds, molecular mechanisms, and therapeutic potential of Mexican Pacific Sargassum species in the context of AMD. Particular emphasis is placed on the modulation of oxidative stress and inflammatory pathways, as well as current translational limitations and future perspectives for the development of marine-derived nutraceutical strategies targeting retinal degeneration.

## 2 Methods

This narrative non-systematic review was conducted through a comprehensive literature search using PubMed, Scopus, Web of Science, and Google Scholar databases to identify relevant studies investigating the bioactive composition, pharmacological properties, and therapeutic potential of Sargassum species in age-related macular degeneration (AMD) and retinal oxidative stress. Search terms included combinations of “Sargassum,” “marine bioactive compounds,” “fucoxanthin,” “fucoidans,” “phlorotannins,” “oxidative stress,” “retinal pigment epithelium,” “inflammation,” “retinal degeneration,” and “age-related macular degeneration.” The review included peer-reviewed articles published between 2010 and 2025, encompassing mechanistic studies, preclinical retinal models, nutraceutical investigations, extraction and bioavailability studies, and relevant review articles. Additional references were identified through manual screening of cited literature. Particular emphasis was placed on studies describing molecular pathways associated with oxidative stress, inflammation, angiogenesis, mitochondrial dysfunction, apoptosis, and retinal protection mediated by Sargassum-derived bioactive compounds.

## 3 Nutritional and bioactive composition of Mexican Pacific Sargassum: polysaccharides, carotenoids, polyphenols, and fatty acids

Brown seaweeds of the genus Sargassum constitute a complex and highly diverse source of biologically active compounds with considerable nutraceutical and pharmacological potential. These marine macroalgae are particularly rich in fucose-containing sulfated polysaccharides (fucoidans), alginates, laminarin/mannitol, phlorotannins (brown algal polyphenols), carotenoids dominated by fucoxanthin, and a modest lipid fraction containing long-chain and intermediate n-3 polyunsaturated fatty acids (PUFAs), the relative abundance of which varies according to species, geographic origin, environmental conditions, seasonal fluctuations, and extraction methodology.

**FIGURE 2:**
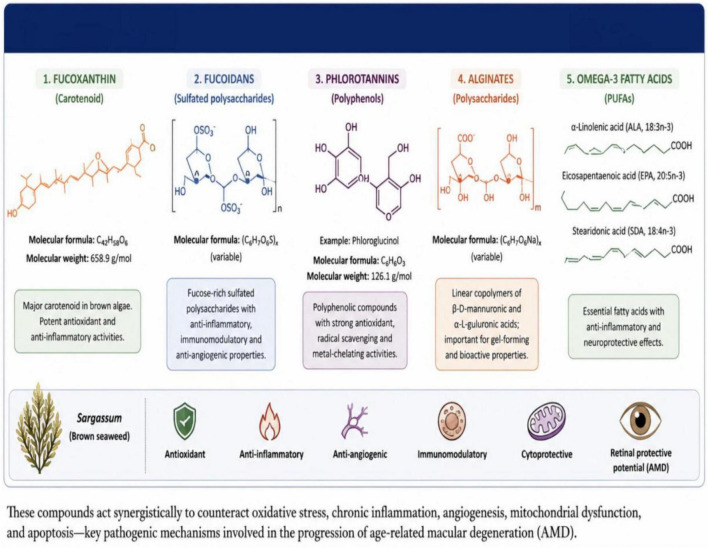
Representative chemical structures of the principal bioactive compounds identified in Mexican Pacific Sargassum species.

Figure 2 illustrates representative chemical structures of the principal bioactive compounds identified in Mexican Pacific Sargassum species.

The structural diversity of Sargassum-derived bioactive compounds is closely associated with their broad spectrum of biological activities and potential therapeutic relevance in retinal degeneration. Fucoxanthin, phlorotannins, fucoidans, alginates, and omega-3 fatty acids possess distinct chemical characteristics that contribute to antioxidant, anti-inflammatory, anti-angiogenic, immunomodulatory, and cytoprotective effects within retinal tissues (14, 18, 21–23). In particular, the presence of sulfated polysaccharides and polyphenolic structures confers strong radical-scavenging and metal-chelating properties, whereas carotenoids such as fucoxanthin exhibit potent photoprotective and redox-regulating activities (11, 12, 18, 24). These multifunctional properties suggest that Sargassum-derived compounds may simultaneously target several interconnected pathogenic mechanisms implicated in AMD, including oxidative stress, mitochondrial dysfunction, inflammatory signaling, apoptosis, and vascular dysregulation (3, 4, 6).

Importantly, the biological activity of these marine-derived compounds is highly influenced by species variability, harvesting season, environmental conditions, extraction methodology, molecular weight distribution, and structural composition (8, 25, 26). Consequently, advanced chemical characterization and extraction standardization remain essential for ensuring reproducibility, bioactivity preservation, and translational applicability in future nutraceutical and pharmacological developments targeting retinal diseases and age-related macular degeneration (21, 27, 28).

Representative proximate ranges reported for *Sargassum* spp. include approximately 14–44% ash, 4–68% carbohydrates, 9–20% protein, and 0.5–3.9% lipids on a dry-weight basis, with carbohydrates primarily composed of alginate and fucoidans. Phlorotannins are taxonomically characteristic metabolites of brown algae belonging to the order Fucales, and their concentration may vary significantly depending on latitude, salinity, ultraviolet exposure, and harvesting season. Fucoxanthin represents the major carotenoid in Sargassum and contributes substantially to its antioxidant and photoprotective properties. Fatty-acid profiles are also highly variable; several Sargassum species contain stearidonic acid (SDA; 18:4n-3) and eicosapentaenoic acid (EPA) associated with glycolipid fractions, although some holopelagic species exhibit comparatively low EPA/DHA concentrations. Studies involving Mexican Caribbean and Pacific Sargassum species, including *S. horridum* and *S*. *sinicola*, demonstrate that biochemical composition strongly depends on environmental and temporal factors, highlighting the importance of species selection and standardization for future nutraceutical applications (8, 13, 16, 19, 23, 25). In addition to their nutritional value, these marine-derived compounds possess significant biological activities directly relevant to retinal degeneration and AMD pathophysiology. Fucoidans exhibit antioxidant, anti-inflammatory, immunomodulatory, and anti-angiogenic properties, with their biological activity strongly influenced by molecular weight, sulfate content, branching patterns, and extraction procedures (14, 21). Phlorotannins are potent radical scavengers and metal-chelating compounds capable of modulating oxidative stress and inflammatory signaling pathways associated with retinal injury. Their structural diversity and bioactivity are influenced by species-specific composition and extraction methodology (11, 18, 19, 24). Fucoxanthin, the characteristic carotenoid of brown algae, demonstrates strong antioxidant, anti-inflammatory, and photoprotective effects, and has also been associated with geroprotective properties through modulation of oxidative stress-related molecular pathways (12, 22, 29). Furthermore, n-3 PUFAs such as SDA and EPA may contribute to retinal protection through modulation of inflammatory mediators, membrane fluidity, and lipid signaling pathways implicated in retinal homeostasis and degeneration (10, 16).

Collectively, the biochemical diversity of Mexican Pacific Sargassum highlights its potential as a multifunctional source of marine-derived bioactive compounds targeting several interconnected pathogenic mechanisms involved in AMD, including oxidative stress, chronic inflammation, mitochondrial dysfunction, angiogenesis, and retinal pigment epithelium degeneration. Nevertheless, substantial variability in chemical composition among species, geographical regions, and harvesting periods remains a major challenge for the development of standardized nutraceutical formulations and translational applications. Consequently, improved chemical characterization, extraction optimization, and molecular standardization are essential to facilitate the future clinical development of Sargassum-derived bioactives for retinal neuroprotection and AMD prevention.

## 4 Mechanisms of action of Sargassum-derived bioactive compounds in AMD: modulation of oxidative stress, inflammation, and apoptosis

**FIGURE 3:**
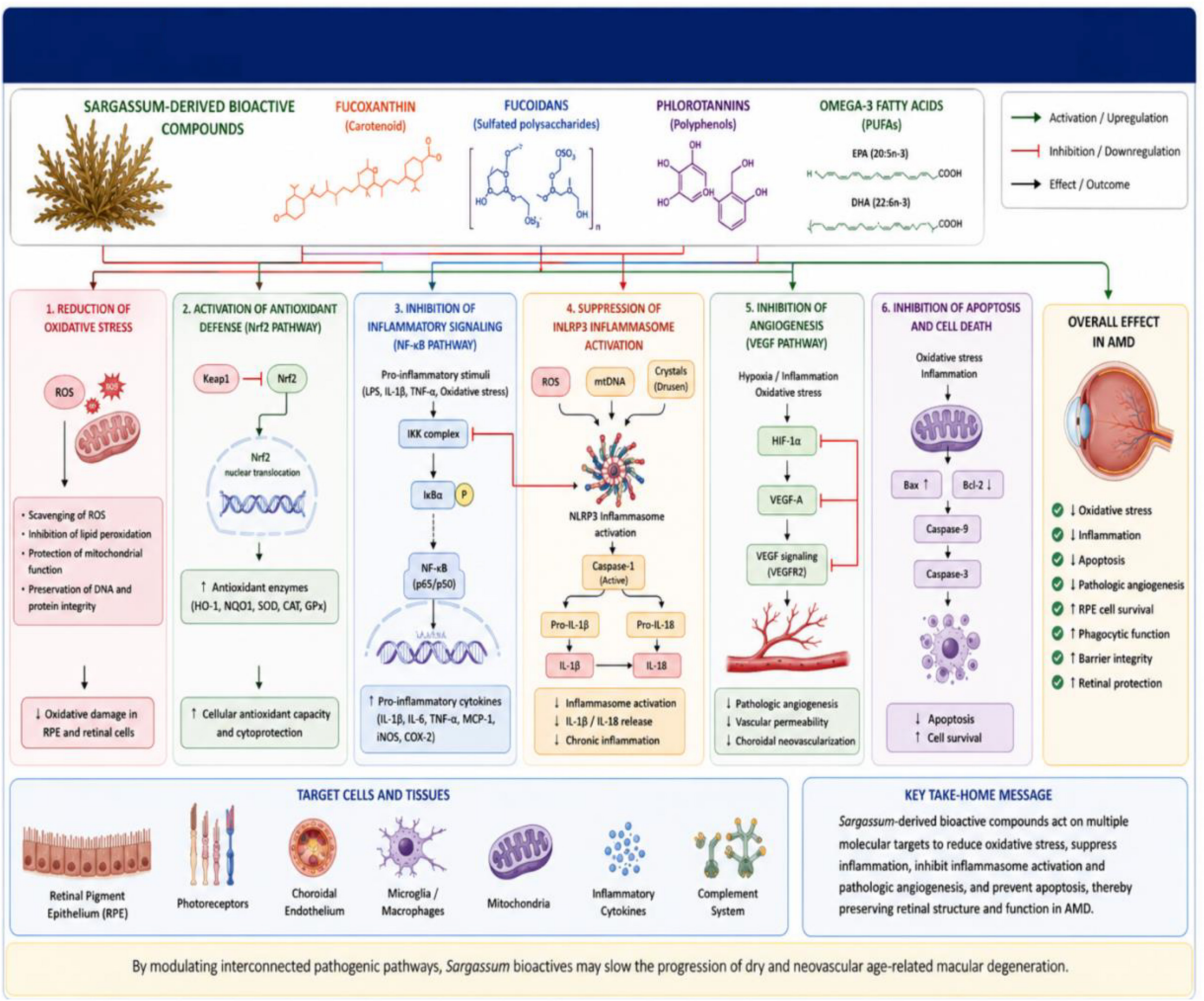
Schematic representation of the mechanisms of Sargassum-derived bioactive compounds in oxidative stress and inflammation.

Age-related macular degeneration (AMD) is driven by a complex interplay of oxidative stress, chronic para-inflammation, mitochondrial dysfunction, complement dysregulation, inflammasome activation, and pathological angiogenesis, all of which contribute to progressive retinal pigment epithelium (RPE) degeneration and photoreceptor loss. Both dry and neovascular AMD are strongly associated with excessive generation of reactive oxygen species (ROS), activation of pro-inflammatory pathways such as NF-κB signaling, complement cascade dysregulation, NLRP3 inflammasome activation, and vascular endothelial growth factor (VEGF)-mediated angiogenesis (Figure 3) (30, 31). Because the retina is characterized by high oxygen consumption, continuous light exposure, and elevated concentrations of polyunsaturated fatty acids, retinal tissues are particularly vulnerable to oxidative injury and mitochondrial dysfunction. Consequently, marine-derived bioactive compounds capable of simultaneously modulating oxidative stress, inflammatory signaling, apoptosis, and angiogenesis have attracted increasing interest as potential adjunctive therapeutic strategies for AMD.

Among the bioactive constituents identified in Sargassum species, fucoxanthin, fucoidans, phlorotannins, and omega-3 fatty acids have received particular attention due to their ability to target multiple interconnected pathogenic pathways implicated in retinal degeneration and AMD progression.

Fucoxanthin (Fx), the principal carotenoid identified in brown algae, exerts potent antioxidant, anti-inflammatory, photoprotective, and cytoprotective effects in retinal cells through modulation of several interconnected molecular pathways. Experimental studies demonstrate that fucoxanthin activates the Nrf2/HO-1 signaling axis, suppresses ROS accumulation, preserves tight junction integrity, maintains phagocytic activity in RPE cells, and protects against visible-light-induced and high-glucose/4-HNE-mediated retinal injury in ARPE-19 and retinal oxidative stress models (15, 17). In addition, fucoxanthin has been associated with geroprotective properties mediated through differential regulation of oxidative stress-responsive genes and mitochondrial signaling pathways (32). Collectively, these mechanisms contribute to reduced oxidative stress, decreased inflammatory mediator production, attenuation of apoptosis-related caspase activation, and preservation of RPE and blood-retinal barrier (BRB) integrity (15). Fucoidans (FUC), sulfated polysaccharides abundant in Sargassum species, exhibit broad anti-inflammatory, immunomodulatory, anti-angiogenic, and antioxidant activities relevant to retinal degeneration. *In vitro* studies demonstrate that fucoidans suppress inflammatory activation in RPE cells, inhibit TLR3-mediated cytokine production, reduce VEGF expression and signaling, and attenuate angiogenic responses associated with choroidal neovascularization (CNV), one of the hallmarks of neovascular AMD (14, 33). In addition to their retinal effects, fucoidans possess antiviral, anti-inflammatory, and antithrombotic properties that are not exclusively dependent on anticoagulant activity, suggesting broader pharmacological potential in chronic inflammatory conditions (34). The biological activity of fucoidans is strongly influenced by molecular weight distribution, sulfate content, extraction methodology, and structural heterogeneity, all of which remain important considerations for translational development.

Phlorotannins (PTs), a characteristic class of brown algal polyphenols, exhibit strong radical-scavenging, metal-chelating, antioxidant, and anti-inflammatory activities. Experimental evidence indicates that phloroglucinol, a representative phlorotannin structural unit, significantly attenuates mitochondrial ROS production, oxidative DNA damage, inflammatory signaling, and apoptosis in ARPE-19 cells exposed to H2O2-induced oxidative stress, supporting the potential role of phlorotannins in retinal protection and mitochondrial preservation (19, 35). Because mitochondrial dysfunction is increasingly recognized as a critical contributor to AMD progression, compounds capable of preserving mitochondrial integrity and limiting oxidative retinal injury may represent promising adjunctive therapeutic strategies.

Polyunsaturated fatty acids (PUFAs) derived from Sargassum lipids, particularly omega-3 fatty acids such as stearidonic acid (SDA) and eicosapentaenoic acid (EPA), may also contribute to retinal homeostasis through modulation of inflammatory mediators, membrane fluidity, lipid signaling pathways, and oxidative stress responses. These compounds may attenuate retinal inflammation by regulating eicosanoid signaling and inflammatory cytokine production. However, despite strong mechanistic rationale and promising preclinical evidence, large AMD clinical trials such as AREDS2 did not demonstrate additional clinical benefit following DHA/EPA supplementation beyond the standard formulation, highlighting the complexity of translating nutritional interventions into measurable clinical outcomes (28).

Overall, the multipronged biological activities of Sargassum-derived bioactive compounds suggest that these marine-derived molecules may simultaneously target several interconnected pathogenic mechanisms involved in AMD progression, including oxidative stress, inflammation, apoptosis, mitochondrial dysfunction, and angiogenesis. Nevertheless, further mechanistic studies, pharmacokinetic investigations, and well-designed clinical trials remain necessary to clarify their translational potential and therapeutic applicability in retinal neurodegeneration and age-related macular degeneration.

**FIGURE 4:**
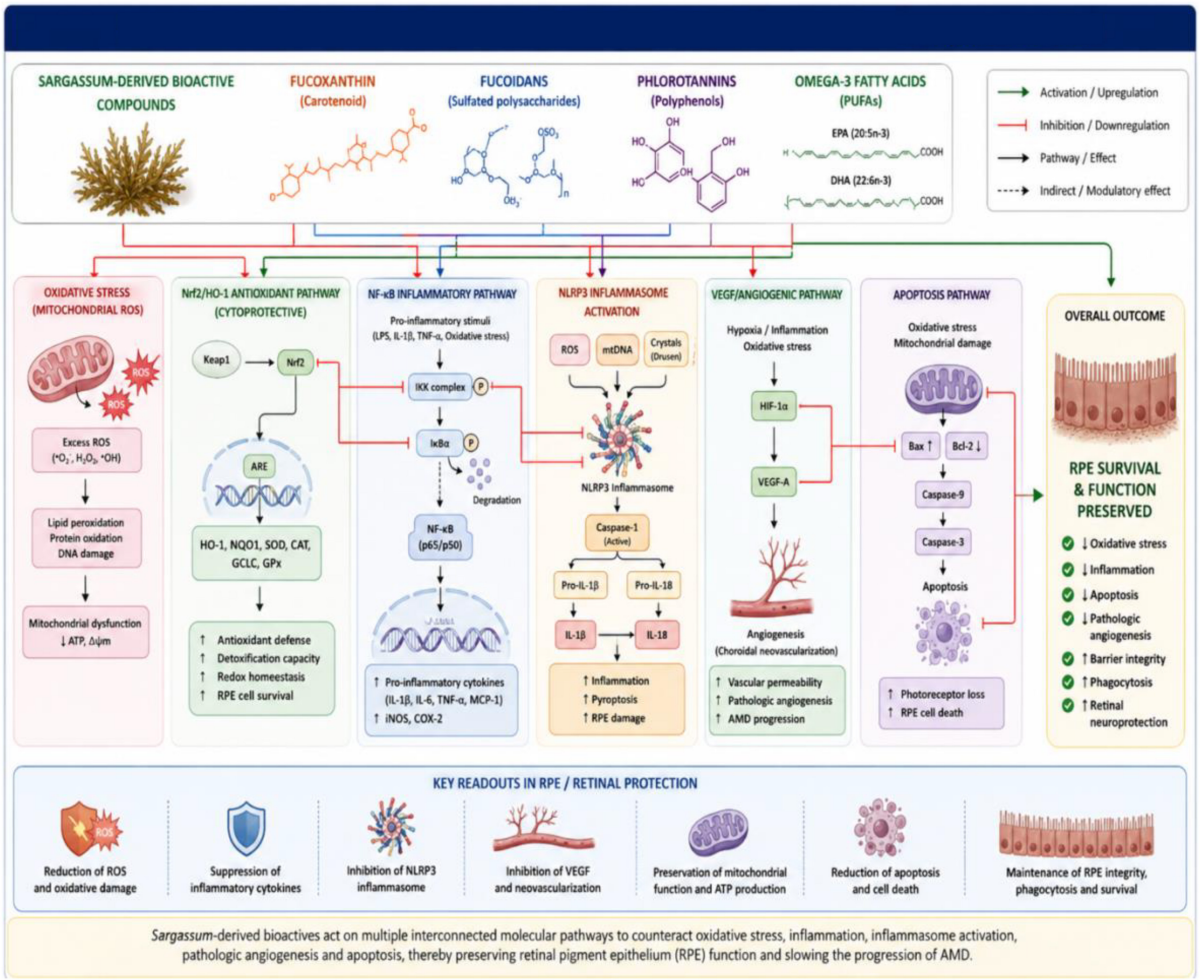
Molecular signaling pathways modulated by Sargassum-derived bioactive compounds in AMD.

Figure 4 illustrates the principal molecular signaling pathways modulated by Sargassum-derived bioactive compounds in AMD, including Nrf2/HO-1 antioxidant signaling, NF-κB-mediated inflammatory activation, NLRP3 inflammasome regulation, VEGF-associated angiogenesis, mitochondrial ROS generation, apoptosis-related pathways, cytokine production, and retinal pigment epithelium survival.

Collectively, these findings support the concept that Sargassum-derived bioactive compounds exert multipronged protective effects through coordinated modulation of oxidative stress, inflammatory signaling, mitochondrial dysfunction, angiogenesis, and apoptosis. By simultaneously targeting several interconnected pathogenic pathways involved in AMD progression, these marine-derived compounds may represent promising adjunctive nutraceutical candidates for retinal neuroprotection and preservation of retinal pigment epithelium function. Nevertheless, additional mechanistic studies, pharmacokinetic investigations, and well-designed clinical trials remain necessary to validate their therapeutic efficacy and translational applicability in age-related macular degeneration.

## 5 Comparative efficacy of Sargassum-derived bioactive compounds versus conventional antioxidants in AMD experimental models

A growing body of experimental evidence suggests that Sargassum-derived bioactive compounds may exert retinal protective effects comparable to, and in certain mechanistic contexts potentially exceeding, those of conventional antioxidant strategies currently used in AMD research and nutritional intervention approaches. In particular, compounds such as fucoxanthin, fucoidans, phlorotannins, and omega-3 fatty acids demonstrate multipronged biological activities involving antioxidant, anti-inflammatory, photoprotective, anti-apoptotic, anti-angiogenic, and mitochondrial-protective mechanisms that directly target key pathogenic pathways implicated in AMD progression.

Table 2 summarizes the comparative biological activities, retinal protective effects, experimental evidence, and translational relevance of Sargassum-derived bioactive compounds relative to conventional antioxidant strategies currently investigated in AMD.

**TABLE 2:** Major bioactive constituents identified in Mexican Pacific Sargassum and their reported biological activities.

Bioactive constituent	Chemical class	Principal biological activities	Potential relevance in AMD	Representative experimental evidence	References
Fucoxanthin	Carotenoid (xanthophyll)	Antioxidant, anti-inflammatory, photoprotective, anti-apoptotic	Reduction of oxidative stress, preservation of RPE integrity, modulation of Nrf2/HO-1 signaling, attenuation of mitochondrial dysfunction	ARPE-19 oxidative stress models; visible-light retinal injury models	(10–12, 22)
Fucoidans	Sulfated polysaccharides	Anti-inflammatory, anti-angiogenic, immunomodulatory, antioxidant	VEGF inhibition, suppression of inflammatory cytokines, reduction of pathological angiogenesis and retinal inflammation	RPE cell models; ocular angiogenesis assays	(13–15)
Phlorotannins	Polyphenolic compounds	Radical scavenging, metal chelation, antioxidant, anti-inflammatory	Protection against oxidative DNA damage, reduction of mitochondrial ROS, anti-apoptotic retinal effects	ARPE-19 oxidative stress and apoptosis models	(16–18, 24)
Alginates	Structural polysaccharides	Gel-forming, antioxidant-supportive, biomaterial applications	Potential use in controlled-release ocular formulations and retinal drug delivery systems	Biomaterial and extraction studies	(21, 26)
DHA and EPA (polyunsaturated fatty acids)	Omega-3 polyunsaturated fatty acids	Anti-inflammatory, antioxidant, neuroprotective, membrane-stabilizing, pro-resolving	Reduce oxidative stress and VEGF signaling, modulate inflammatory cytokines and retinal microglial activation, preserve photoreceptor and RPE integrity, and support retinal homeostasis in AMD	DHA/EPA supplementation reduces inflammatory mediators, protects retinal cells from oxidative damage, and supports retinal structure and function in AMD models	(36–39)
Laminarin	β-glucan polysaccharide	Antioxidant, immunomodulatory	Potential modulation of oxidative stress and inflammatory signaling pathways	Preclinical marine polysaccharide studies	(23, 25)
Mannitol	Sugar alcohol/polyol	Osmoprotective and antioxidant-supportive properties	Potential reduction of oxidative cellular stress	Marine algal biochemical studies	(8, 23)
Polyphenolic extracts	Mixed phenolic compounds	Antioxidant, anti-inflammatory, cytoprotective	Reduction of lipid peroxidation and inflammatory retinal injury	Experimental antioxidant and retinal stress models	(7, 21)

Preclinical investigations indicate that fucoxanthin consistently exhibits strong antioxidative and cytoprotective effects in retinal pigment epithelium (RPE) models exposed to oxidative stress. Experimental studies demonstrate that fucoxanthin suppresses ROS accumulation, preserves mitochondrial integrity, attenuates apoptosis-related signaling, and modulates Nrf2/HO-1 antioxidant pathways in ARPE-19 cells and retinal oxidative injury models (15, 17, 35). In several *in vitro* and experimental settings, fucoxanthin-mediated protection appears comparable or superior to conventional antioxidant reference compounds, depending on dosage, experimental conditions, and evaluated endpoints. Similarly, fucoidans demonstrate potent anti-inflammatory and anti-angiogenic effects through suppression of VEGF signaling, inflammatory cytokine production, and pathological angiogenesis associated with choroidal neovascularization (14, 33). Phlorotannins further contribute to retinal protection through radical-scavenging activity, mitochondrial ROS suppression, and reduction of oxidative DNA damage and apoptosis in retinal cells (17, 35).

By contrast, conventional carotenoids such as lutein and zeaxanthin remain the only nutraceutical interventions with substantial outcome-level evidence in AMD clinical management, particularly through the AREDS2 formulation. Replacement of β-carotene with lutein/zeaxanthin was associated with improved safety and additional reduction in progression to late AMD, supporting their role as current clinical standards in nutritional retinal support (19). However, despite strong mechanistic rationale, supplementation with long-chain omega-3 fatty acids did not demonstrate additional clinical benefit in AREDS2 beyond standard antioxidant therapy, underscoring the complexity of translating preclinical antioxidant effects into measurable clinical outcomes (28, 40, 41).

Importantly, Sargassum-derived compounds differ from conventional antioxidant strategies because they exert simultaneous effects on multiple interconnected molecular pathways implicated in AMD, including oxidative stress, inflammation, angiogenesis, mitochondrial dysfunction, apoptosis, and retinal pigment epithelium degeneration. This multitarget pharmacological profile may provide theoretical advantages over single-pathway antioxidant approaches, particularly in multifactorial retinal diseases characterized by complex molecular crosstalk. Nevertheless, interpretation of current evidence should be approached cautiously because most available data remain restricted to *in vitro* studies, experimental retinal models, and preclinical investigations.

Despite promising mechanistic and experimental findings, no AMD-specific randomized clinical trials have yet validated the efficacy of fucoxanthin, fucoidans, or phlorotannins in human retinal disease. Consequently, although Sargassum-derived bioactive compounds represent highly promising nutraceutical candidates with substantial translational potential, further pharmacokinetic studies, formulation optimization, standardized extraction procedures, and well-designed clinical trials remain necessary before these compounds can be incorporated into evidence-based therapeutic strategies for age-related macular degeneration.

## 6 Challenges related to bioavailability, extraction methods, and standardization of dosages

Despite the growing body of experimental evidence supporting the antioxidant, anti-inflammatory, anti-angiogenic, and cytoprotective properties of Sargassum-derived bioactive compounds, several critical translational limitations continue to hinder their development as evidence-based nutraceutical or pharmacological interventions for age-related macular degeneration (AMD). Among the most important challenges are low oral bioavailability, limited pharmacokinetic characterization, extraction variability, structural heterogeneity, inadequate dosage standardization, and the absence of AMD-specific clinical trials. Because the biological activity of marine-derived compounds is highly dependent on molecular structure, extraction methodology, metabolic transformation, and formulation stability, optimization of these parameters remains essential for successful clinical translation.

Bioavailability and metabolism represent major barriers for several Sargassum-derived compounds. Fucoxanthin, a highly lipophilic carotenoid, undergoes rapid deacetylation in the gastrointestinal tract to fucoxanthinol and subsequent conversion to amarouciaxanthin A in peripheral tissues. Although these metabolites may retain biological activity, the low aqueous solubility and limited systemic bioavailability of fucoxanthin substantially restrict its translational applicability. Recent investigations demonstrate that emulsification technologies, lipid-based formulations, nanoemulsions, and colloidal delivery systems may significantly improve intestinal absorption and bioaccessibility (42). Human studies outside the ophthalmological context have generally evaluated fucoxanthin doses ranging from approximately 2–8 mg/day, although optimal therapeutic concentrations for retinal protection remain unknown.

Phlorotannins also present important pharmacokinetic limitations due to their large molecular size, polymeric structure, and considerable chemical heterogeneity. Their gastrointestinal absorption is relatively limited and strongly dependent on molecular complexity, degree of polymerization, and structural composition. Emerging pharmacokinetic studies involving phlorotannins from species such as Ecklonia cava demonstrate measurable but highly variable oral bioavailability, indicating the need for improved formulation strategies and more detailed metabolic characterization (18). Similarly, fucoidans exhibit poor intestinal absorption when present as high-molecular-weight polysaccharides. Enzymatic depolymerization and controlled hydrolysis generating low-molecular-weight fucoidan derivatives have demonstrated improved systemic uptake and potentially enhanced biological activity, highlighting molecular size as a critical determinant of bioavailability and pharmacological efficacy (27, 43, 44).

Extraction methodology and chemical standardization also represent major sources of variability influencing biological activity, reproducibility, and translational development. For fucoxanthin, supercritical CO2 extraction combined with ethanol co-solvents and advanced green extraction technologies, including microwave-assisted extraction (MAE), ultrasound-assisted extraction (UAE), and ionic liquid-based approaches, have demonstrated improved extraction efficiency, compound purity, and preservation of structural stability (11, 45–48). However, extraction conditions may substantially affect isomerization, oxidation, and biological activity of carotenoid fractions. In the case of phlorotannins, contemporary extraction technologies have improved yield and antioxidant potency compared with conventional solvent extraction methods, yet structural characterization and reproducibility remain technically challenging because of their high chemical diversity and polymeric complexity (21, 49–51).

Fucoidan extraction and purification present additional technical challenges because conventional hot-acid extraction procedures frequently co-isolate alginates and other polysaccharides, complicating structural characterization and standardization. More recent enzyme-assisted and multistage extraction approaches permit generation of structurally preserved fucoidans with tunable molecular weight and sulfation profiles, potentially improving reproducibility and pharmacological consistency (21, 26, 52). Nevertheless, substantial variability in sulfate content, branching patterns, molecular size, and geographical origin continues to complicate comparison among experimental studies.

The principal translational challenges associated with Sargassum-derived bioactive compounds, including bioavailability limitations, extraction variability, molecular standardization, and safety considerations, are summarized in Table 3.

**TABLE 3:** Challenges and translational considerations for Sargassum-derived bioactives in age-related macular degeneration (AMD).

Translational challenge	Major limitation	Potential impact on AMD application	Proposed strategies/solutions	Representative evidence	References
Low bioavailability of fucoxanthin	Poor aqueous solubility, rapid metabolism to fucoxanthinol and amarouciaxanthin A	Reduced systemic and retinal bioavailability, limited therapeutic efficacy	Nanoemulsions, lipid carriers, emulsification systems, phospholipid complexes	Improved intestinal absorption and bioaccessibility reported in experimental formulations	(11, 42)
Limited oral absorption of phlorotannins	Large polymeric structure and structural heterogeneity	Variable gastrointestinal absorption and inconsistent pharmacokinetics	Structural optimization, advanced delivery systems, molecular characterization	Variable but measurable oral bioavailability in marine polyphenol studies	(18, 51)
Poor absorption of high-molecular-weight fucoidans	High molecular weight and sulfate-dependent variability	Reduced systemic exposure and translational applicability	Controlled depolymerization, low-MW fucoidan derivatives, enzymatic processing	Improved uptake and biological activity with low-MW fractions	(27, 43, 44)
Extraction variability	Species-, season-, and method-dependent variability in yield and composition	Inconsistent bioactivity and poor reproducibility among studies	Standardized extraction protocols, green extraction technologies, quality control systems	MAE, UAE, and supercritical CO2 extraction improve yield and purity	(45–48)
Structural heterogeneity of phlorotannins and fucoidans	Variability in sulfation degree, branching, molecular size, and composition	Difficulty comparing experimental outcomes and establishing dosage consistency	Advanced analytical characterization (HPLC, LC-MS/MS, NMR, FT-IR)	Structural variability strongly influences biological activity	(21, 24, 26)
Limited retinal pharmacokinetic data	Scarcity of ocular distribution and retinal tissue availability studies	Uncertain therapeutic retinal concentrations and dosing regimens	Pharmacokinetic and biodistribution studies in retinal models	Lack of AMD-specific PK data remains a major limitation	(22, 28)
Safety and toxicological concerns	Potential accumulation of heavy metals (As, Cd, Pb, Hg) in Sargassum biomass	Toxicity risk and regulatory limitations for nutraceutical development	Heavy-metal screening, toxicological assessment, GMP compliance	Marine algae may bioaccumulate environmental contaminants	(33, 54, 56)
Lack of AMD-specific clinical trials	Evidence largely restricted to preclinical retinal models	Limited clinical validation and translational applicability	Randomized clinical trials, standardized formulations, retinal outcome measures	No AMD-specific randomized trials available for Fx/FUC/PTs	(10, 14, 17)
Absence of dosage standardization	Variable purity, extraction conditions, and molecular composition	Poor reproducibility and uncertain therapeutic efficacy	Standardization using molecular markers (% fucoxanthin, MW, sulfation degree)	Molecular standardization essential for translational development	(21, 26, 27)
Regulatory and manufacturing challenges	Incomplete quality-control frameworks for marine nutraceuticals	Delayed clinical translation and commercialization	GMP implementation, validated analytical methods, regulatory harmonization	Quality-control and safety evaluation required for clinical development	(28, 42, 55)

Another important limitation involves the absence of standardized dosage regimens and the scarcity of human clinical studies specifically focused on AMD. Currently, no evidence-based therapeutic dose has been established for fucoxanthin, phlorotannins, or fucoidans in retinal diseases. Available non-ocular human studies suggest that fucoidan supplementation at approximately 300 mg/day is generally well tolerated, while phlorotannin-rich preparations appear relatively safe at doses up to approximately 500 mg/day for periods ranging from 8 to 12 weeks. However, the relationship between systemic exposure, retinal tissue bioavailability, and clinical efficacy remains poorly understood. Importantly, all formulations intended for nutraceutical development should be rigorously standardized according to active molecular markers, including fucoxanthin concentration, fucoidan molecular weight and sulfation degree, and total or individual phlorotannin content.

Safety and quality control are also critical considerations because Sargassum species possess a strong capacity to accumulate environmental contaminants, including heavy metals such as arsenic (As), cadmium (Cd), lead (Pb), and mercury (Hg). Consequently, rigorous toxicological evaluation, contaminant monitoring, and compliance with pharmacopeial quality standards are indispensable prerequisites for future clinical applications and regulatory approval (28, 42, 53–56).

**FIGURE 5:**
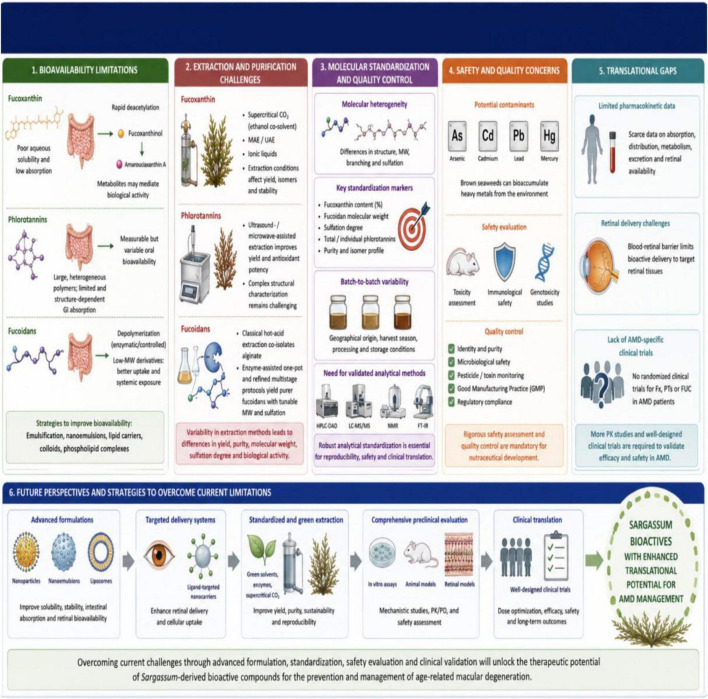
Challenges and translational limitations of Sargassum-derived bioactive compounds in AMD and future perspectives for clinical development.

Figure 5 illustrates the major translational barriers and future perspectives associated with the development of Sargassum-derived bioactive compounds for AMD, including limitations related to bioavailability, extraction technologies, molecular standardization, safety evaluation, retinal delivery, and clinical validation.

Collectively, these limitations indicate that although Sargassum-derived bioactive compounds possess substantial therapeutic potential in retinal degeneration and AMD, major advances in extraction technology, formulation science, pharmacokinetic characterization, molecular standardization, and clinical validation are still required before these marine-derived compounds can be translated into evidence-based nutraceutical interventions for ophthalmological applications.

## 7 Current state and future perspectives: preclinical evidence, clinical applicability, translational limitations, and future directions

Accumulating experimental evidence strongly supports the therapeutic potential of Sargassum-derived bioactive compounds as multifunctional modulators of oxidative stress, inflammation, angiogenesis, mitochondrial dysfunction, and apoptosis in age-related macular degeneration (AMD). Among these compounds, fucoxanthin (Fx), fucoidans (FUC), and phlorotannins (PTs) have demonstrated particularly promising biological activities in retinal experimental models, suggesting that marine-derived bioactives may represent emerging nutraceutical candidates for retinal neuroprotection and preservation of retinal pigment epithelium (RPE) integrity. Nevertheless, despite increasingly robust mechanistic evidence, substantial translational limitations continue to prevent their incorporation into evidence-based clinical strategies for AMD management.

Preclinical investigations indicate that marine brown-algal constituents abundant in Sargassum species exert disease-relevant protective effects across multiple interconnected pathogenic pathways implicated in retinal degeneration. Fucoxanthin activates the Nrf2/HO-1 antioxidant signaling pathway, suppresses mitochondrial ROS generation, attenuates oxidative injury, and reduces apoptosis in retinal cells exposed to oxidative stress conditions.

In ARPE-19 models, fucoxanthin additionally preserves tight-junction integrity, maintains phagocytic function, and mitigates visible-light-induced retinal damage, collectively supporting its cytoprotective and mitochondrial-preserving properties (10, 11). Fucoidans demonstrate potent anti-inflammatory and anti-angiogenic activities through suppression of VEGF signaling, inflammatory mediator production, and pathological angiogenesis associated with choroidal neovascularization (13, 14). Similarly, phlorotannins and phloroglucinol-derived compounds reduce oxidative DNA damage, mitochondrial dysfunction, and apoptosis in retinal cells subjected to oxidative stress, consistent with their strong radical-scavenging and anti-inflammatory capacities (16, 17). Collectively, these findings support the concept that Sargassum-derived bioactives act as multipronged regulators of oxidative stress, para-inflammation, mitochondrial dysfunction, and retinal cell survival in AMD pathophysiology (10, 11, 14, 17).

Despite these promising mechanistic observations, clinical applicability remains limited because current evidence is still predominantly restricted to preclinical and formulation-oriented investigations. Conventional carotenoids such as lutein and zeaxanthin remain the only nutraceutical interventions with substantial outcome-level evidence in AMD management through the AREDS2 formulation. Although replacement of β-carotene with lutein/zeaxanthin improved safety and reduced progression toward late AMD, supplementation with long-chain omega-3 polyunsaturated fatty acids did not demonstrate additional clinical benefit in AREDS2, underscoring the complexity of translating nutritional interventions into measurable ophthalmological outcomes (28, 40, 41). In contrast, no AMD-specific randomized clinical trials have yet validated the efficacy of fucoxanthin, fucoidans, or phlorotannins in retinal disease, and current support remains mechanistic and experimental rather than clinically validated (10, 11, 14, 17, 27). Consequently, Sargassum-derived compounds should currently be regarded as promising adjunctive nutraceutical candidates rather than evidence-based replacements for established AMD interventions.

Several major translational barriers continue to limit the clinical development of Sargassum-derived bioactive compounds. One of the most important limitations involves poor oral bioavailability and limited pharmacokinetic characterization. Fucoxanthin exhibits low aqueous solubility and undergoes rapid metabolic transformation into fucoxanthinol and amarouciaxanthin A, whereas high-molecular-weight fucoidans demonstrate poor intestinal absorption and limited systemic exposure. Phlorotannin bioavailability also remains highly variable and strongly dependent on molecular structure and degree of polymerization (11, 16, 22, 24). In addition, extraction methodology, molecular heterogeneity, sulfation degree, environmental variability, and species-dependent biochemical composition substantially influence biological activity and reproducibility among studies (24, 27, 28, 35). These factors complicate standardization, dose optimization, and translational comparability, representing major obstacles for future clinical development.

Safety and quality control also represent critical considerations because Sargassum species possess a strong capacity to accumulate environmental contaminants, including arsenic, cadmium, lead, and mercury. Therefore, rigorous toxicological assessment, contaminant monitoring, pharmacopeial standardization, and Good Manufacturing Practice (GMP) compliance are essential prerequisites for future nutraceutical and pharmacological applications (33). Furthermore, the absence of retinal pharmacokinetic studies, standardized formulations, validated retinal biomarkers, and AMD-specific clinical endpoints significantly hinders translational progress and therapeutic validation (10, 15, 17, 19, 22, 30).

Future research should therefore prioritize the development of standardized and chemically characterized Sargassum-derived formulations, including detailed characterization of fucoidan molecular weight and sulfation patterns, fucoxanthin isomerization profiles, and phlorotannin composition. Additional efforts should focus on advanced formulation strategies capable of improving retinal bioavailability, including nanoemulsions, lipid-based delivery systems, phospholipid carriers, and targeted nanotechnological platforms (27, 42–44, 57). Comparative preclinical investigations evaluating Sargassum-derived compounds against conventional antioxidant therapies under standardized oxidative and inflammatory retinal conditions are also necessary to better define therapeutic efficacy and mechanistic advantages (15, 17, 33, 35, 41).

From a translational perspective, future clinical development should include well-designed randomized clinical trials in intermediate AMD populations using standardized Sargassum-derived formulations as adjunctive nutraceutical interventions. Potential endpoints should include macular pigment optical density (MPOD), drusen volume, dark adaptation kinetics, inflammatory biomarkers, retinal imaging parameters, and visual function outcomes (28, 41). Simultaneously, sustainable harvesting strategies and geo-chemical characterization of Mexican Pacific Sargassum species should be implemented to ensure bioactive consistency, environmental sustainability, and quality control throughout future industrial development (8, 20, 27, 28).

Overall, the available evidence indicates that Sargassum-derived bioactive compounds possess substantial mechanistic and translational potential for targeting multiple interconnected pathogenic pathways involved in AMD progression. Although current evidence remains predominantly preclinical, continued advances in extraction technologies, formulation science, pharmacokinetics, molecular standardization, and clinical validation may ultimately facilitate the development of innovative marine-derived nutraceutical strategies for retinal neuroprotection and age-related macular degeneration management.

## 8 Conclusion

Marine-derived bioactive compounds obtained from Mexican Pacific Sargassum species represent a highly promising and still underexplored source of multifunctional nutraceutical candidates for age-related macular degeneration (AMD). The growing body of experimental evidence reviewed in this study indicates that fucoxanthin, fucoidans, phlorotannins, alginates, and omega-3 fatty acids exert antioxidant, anti-inflammatory, anti-angiogenic, mitochondrial-protective, and cytoprotective effects that directly target several interconnected pathogenic mechanisms implicated in retinal degeneration. Through modulation of molecular pathways associated with oxidative stress, NF-κB-mediated inflammation, Nrf2/HO-1 signaling, VEGF-associated angiogenesis, mitochondrial dysfunction, inflammasome activation, and apoptosis, these marine-derived compounds demonstrate considerable therapeutic potential for preserving retinal pigment epithelium integrity and retinal homeostasis.

Importantly, the multitarget biological activity of Sargassum-derived compounds may provide theoretical advantages over conventional single-pathway antioxidant strategies currently employed in AMD management. Nevertheless, despite increasingly robust mechanistic and preclinical evidence, substantial translational barriers continue to limit clinical applicability. Variability in chemical composition, limited oral bioavailability, insufficient pharmacokinetic characterization, extraction heterogeneity, molecular standardization challenges, and the absence of AMD-specific clinical trials remain major obstacles for therapeutic development and regulatory implementation.

Future investigations should therefore prioritize standardized extraction methodologies, advanced formulation technologies, retinal-targeted delivery systems, comprehensive pharmacokinetic analyses, and well-designed randomized clinical trials capable of validating efficacy, safety, and long-term retinal outcomes in AMD patients. In parallel, sustainable harvesting practices and rigorous quality-control strategies will be essential to ensure reproducibility, environmental sustainability, and translational feasibility of Sargassum-derived formulations.

Overall, Mexican Pacific Sargassum represents a strategically important marine bioresource with significant potential for the development of next-generation nutraceutical and adjunctive therapeutic approaches targeting oxidative stress, chronic inflammation, mitochondrial dysfunction, and retinal neurodegeneration in age-related macular degeneration.
